# Effects of dietary diversification during pregnancy on birth outcomes in east Gojjam, northwest Ethiopia: A prospective cohort study

**DOI:** 10.3389/fpubh.2022.1037714

**Published:** 2022-12-05

**Authors:** Amsalu Taye Wondemagegn, Binalfew Tsehay, Akiloge Lake Mebiratie, Ayenew Negesse

**Affiliations:** ^1^Department of Biomedical Sciences, School of Medicine, Debre Markos University, Debre Markos, Ethiopia; ^2^Department of Obstetrics and Gynecology, School of Medicine, Debre Markos University, Debre Markos, Ethiopia; ^3^Department of Human Nutrition and Food Sciences, Health Science College, Debre Markos University, Debre Markos, Ethiopia

**Keywords:** dietary diversity, pregnancy outcomes, low birth weight, preterm birth, still birth, fetal death, Ethiopia

## Abstract

**Introduction:**

Adequate nutrient intake during pregnancy is an important key factor affecting fetal growth and birth outcomes, as well as maternal health, as demonstrated by experimental animal studies. However, the few human studies available, especially those conducted in the least developed countries (LDCs), are much less consistent. Therefore, this study aimed to investigate the association between dietary diversification during pregnancy and birth outcomes in Gojjam, Ethiopia.

**Methodology:**

A facility-based prospective cohort study was conducted on 416 pregnant mothers (exposed and non-exposed) from December 2019 to January 2020. Information about the Women's Dietary Diversity Score (WDDS) was collected using the Food and Agricultural Organization's guidelines. Data were collected through interviewer-administered questionnaires and measurements. Log-binomial regression analysis was performed to estimate the relative risk of adverse birth outcomes. Energy, macronutrient, and micronutrient amounts were calculated using the nutrition survey software version 2007. The differences between groups were noticed using analysis of variance. Eta squared was estimated in the current study.

**Results:**

The overall magnitude of low birth weight, preterm birth, and stillbirth in the study area, respectively, was 41%, 38%, and 4%. After adjustment for baseline characteristics, this study revealed that pregnant women in the inadequate WDDS group were at increased risk of LBW (ARR = 6.4; 95% CI: 3.4, 12) and PTD (ARR = 6.3; 95% CI: 3.3, 11.95) as compared with their counterparts but no difference in the occurrence of stillbirth (ARR = 1.08; 95% CI: 0.20, 5.79).

**Conclusion:**

Overall, this study found a large magnitude of low birth weight and preterm birth. Inadequate intake of dietary diversity during pregnancy significantly increased the rate of low birth weight and preterm birth. Thus, we recommend the concerned body to work on improving the feeding practices of pregnant mothers in the study area.

## Introduction

Dietary diversity is defined as the consumption of many different foods or food groups over a period, most often in the past 24 h or in a week (1, 2). According to a study (3), adequate nutrient intake necessary for good nutrition has often been associated with food variety and the quality of the diet. Unfortunately, literature shows that micronutrient malnutrition remains a major public health concern in developing countries due to the intake of repetitive, predominantly starchy-based diets that are lacking in diversity (4). In addition, pregnant women in these countries are considered the most vulnerable since they have higher nutrient requirements (5).

Documented experimental animal studies and studies conducted on humans in developed countries revealed that nutrient intake during gestation is an important factor influencing fetal growth and birth outcomes as well as the health of the mother (6–9). Sufficient nutrient consumption is a vital event during pregnancy for healthy birth outcomes and for gaining normal health of the mother (10). On the other hand, deprived nutritional status during pregnancy has been associated with adverse birth outcomes such as low birth weight babies, intrauterine growth retardation, and preterm birth (9, 11).

In contrast to the above-mentioned evidence conducted on animals and humans in developed countries, the very few existing studies conducted on humans in the least developed countries came up with varying findings showing positive associations and no association (12–15). This controversy might be because previous studies that revealed the association between maternal nutrient intake and adverse birth outcomes have been approached through the use of a single nutrient intake (7). This approach is not appropriate because, in socio-economically deprived populations, such as Ethiopia, multiple nutrient deficiencies are more likely to occur than single deficiencies (16). An indicator that can reveal multiple nutrient deficiencies will best measure the overall adequacy of nutrient intake in these settings. Quantitative assessment of food intake has always been a challenging task. This is more in the populations where food is eaten from a common container, and individual intake is not easily quantified (17).

Available scientific evidence suggests that dietary diversity scores can be a measure of the diet adequacy of women (18). The concept of dietary diversity score (DDS) in diet quality assessment has been tried in many places among some population groups (19, 20). This approach places emphasis on a non-quantitative assessment of actual food consumption. Dietary diversity score (DDS) is relatively quite simple to apply and it has been shown to reflect micronutrient intake (20).

With the best of our efforts, we paid little attention to observing the effects of multiple nutrient deficiencies on birth outcomes such as LBW, preterm delivery, fetal death, and neonatal health, especially in poor socioeconomic settings such as Ethiopia. Thus, the present study will fill this gap. As a result, a prospective cohort study was conducted to investigate the association between dietary diversity during pregnancy and adverse pregnancy outcomes using the food and agricultural organization's (FAO's) guidelines (21, 22) to collect information about the Women's Dietary Diversity Score (WDDS). Women's dietary diversity scores are valid indicators of nutrient adequacy of the diets of women (1, 3, 18), which in turn has a determinative role in birth outcomes. Thus, women were categorized into adequate (high) and inadequate (low) dietary diversity groups, based on the level of the food groups consumed. Hence, the study aimed to investigate the association between dietary diversification during pregnancy and birth outcomes in Gojjam, northwest Ethiopia, 2019/2020. Clarifying the association between dietary diversity and birth outcomes prospectively will enable healthcare providers to provide evidence-based health education for pregnant mothers at their healthcare delivery ward. In addition, knowing the association between diet diversity and birth outcomes will enable the local healthcare planners and policymakers to design strategies for improving the feeding practices of pregnant mothers in the study area.

## Methodology

### Study area and period

The study was conducted in East Gojjam, Amhara regional state from December 2019 to January 2020. East Gojjam administrative zone has 18 woredas; in which Debre Markos town is the capital of the zone, which is 299 km from the capital, Addis Ababa, and 256 kmfrom the regional state capital, Bahir Dar. According to the 2007 Ethiopia population and housing census (23), the zone has a total population of 2,153,937, of whom 1,066,716 are men and 1,087,221 are women, with an area of 14,004.47 square kilometers. In this zone, there are currently 10 hospitals, 102 health centers, and 423 health posts (24). The zone has different agroecology. According to the reports of the zone's agricultural offices, the altitude ranged from 1,500 to 3,577 m above sea level. The average annual rainfall varied from 900 to 2,000 mm, and the average minimum and maximum temperatures ranged from 7 to 15 and 22–25°C, respectively.

### Study design

An institution-based prospective cohort study was conducted using pregnant mothers who had taken inadequate nutrients (WDDS < 5) as an exposure group and those who had taken adequate nutrients (WDDS ≥ 5) (22) and was considered as a control group.

### Sample size and sampling procedures

The source population for the study was all permanent (living 6 months or above) residents in the area who had confirmed pregnancy and had confirmed to stay in the area until delivery of the newborn. The sample size was calculated using double population proportion determination formula based on the assumption of a 95% confidence interval, 80% power, 5% margin of error, and one-to-one ratio, using the proportion of low birth weight among the exposed group, and 12.45% and 5.85% in unexposed groups (12). The final sample size after adding 15% for loss to follow-up is 416 (208 exposed and 208 unexposed).

To get the study participants, a multistage sampling technique was used. First, from the zone, 50% of health institutions were selected, then in each health institution, the sampling frames were prepared. The sampling frames for the control and exposed groups were different.

### Recruitment and allocation

Participants in their first and second trimesters were selected by simple-random sampling technique at the selected health institutions. The gestational age of the fetus was determined by the date given by the participants as their last menstrual period (LMP). Only those who meet the inclusion criteria were enrolled (i.e., those living in the area for 6 months and above). The exclusion criteria for the study participants were as follows: pregnant women with known medical, surgical, obstetric, and mental problems. In addition, a pregnant woman was assigned and remained in an adequate or inadequate group if her WDDS remained in that category for greater or equal to three of the four visits. Otherwise, the woman was excluded from the analysis.

### Data collections

#### Data collection instruments and data collectors

Data collection tools were developed by reviewing different literature. Translation to the local language, Amharic, and back translation of the tool was done to maintain consistency. A pre-test of the tool in 5% of the sample size was conducted before the actual data collection and errors identified were corrected. All data collectors were experienced health professionals, like midwives or nurses with at least a qualified diploma. In each selected health center, one supervisor was assigned to oversee data collection. In addition, the investigators made a weekly visit to check the completeness and quality of the data that was collected.

### Data collection procedure

#### General information

Socio-demographic and general data were collected through an interviewer-administered structured questionnaire.

#### Dietary intake assessment

A 24-h food recall was conducted to measure the diet diversity and nutrient adequacy of study subjects. Participants were asked for a complete list of all items used in the preparation of meals. All food items and drinks consumed on the previous day were recorded, using a 24-h recall. Local household food-preparing utensils including glasses, spoons, cups, and tableware were considered for estimating the number of foods and drinks consumed by the study participants. These local utensils served as a visual aid to enhance the precision of portion size estimations. Dietary information was collected three times and the mean was computed for nutrient estimations. The information on individual food items and portion sizes was then analyzed to estimate nutrient adequacy. The collected homegrown and homemade foods consumed by each study participant were synchronized with a United States Department of Agriculture (USDA) food composition table considering the nutritional equivalent (25). Collected food data of different sizes and compositions were first converted into gram equivalents and then energy, macronutrient, and micronutrient amounts were calculated using the nutrition survey software version 2007 [professional German nutrition software (EBISpro)].

#### Minimum DD-women indicator

DD for this study was calculated using the minimum DD-women (MDD-W) indicator, which is an improved version of the WDD score which considers 10 food groups, and consumption of at least five of which indicates high DD groups (22). The 10 food groups are as follows: (1) Grains, white roots and tubers, and plantains; (2) Pulses (beans, peas, and lentils); (3) Nuts and seeds; (4) Dairy; (5) Meat, poultry, and fish; (6) Eggs; (7) Dark green leafy vegetables; (8) Other vitamin A-rich fruits and vegetables; (9) Other vegetables; and (10) Other fruits. Dietary diversity was measured three times using a 24-h food recall and the average of the three scores for all women was used to define low and high DD. For each participant, the consumption of at least five food groups in 24 h was given 1 point, and if not consumed at least five food groups were given 0 points. For each study participant, a minimum of 0 and a maximum of 10 points could be gained. Higher scores direct higher diversity, as more food groups were described to be consumed. In addition, participants consuming at least 20 grams/day from each food group were coded as 1 and those consuming less than 20 grams/day from each food group were coded as 0. A higher score indicates a better quality of diet consumed, as consumption from more food groups may provide a variety of nutrients that probably may not be fulfilled by consumption of limited food groups.

#### Measurement of pregnancy outcomes

The pregnancy outcomes record was obtained from the physician's notes after delivery.

#### Follow-up

There were three follow-up visits after recruitment. Two were during pregnancy and the third was after delivery. Socio-demographic and general information was collected at recruitment. Dietary intake was measured at recruitment and the first two follow-up visits. Individuals who dropped-out/lost to follow-up/transferred out or died of any disease during the study period were excluded from the analysis.

### Data processing and analysis

Each questionnaire was given a code and entered into the Epi-Data version 3.1 statistical package and exported to the SPSS version 20 statistical package for analysis. Before analysis, data cleaning using frequency, listing, and sorting was done to identify any outliers and missed values, and then corrections were made by revising the original questionnaire. In addition, multi-colinearity was checked using the tolerance/variance inflation factor. Descriptive results were presented using percentage, frequency, and graphs for categorical variables, and continuous variable data were presented as means plus or minus standard deviations (SD). The differences between groups were noticed using one-way ANOVA and Eta squared was calculated to estimate the measure of association and presented as proportion. The risk ratio was used for presenting inferential statistics. An independent sample *t*-test was used to compare the means of dependent variables in the adequate and inadequate groups. A Chi-square test or Fisher's exact test was used to test for independence in the distribution of categorical variables between the two study groups. Log-binomial regression analysis was performed to estimate the relative risk with a corresponding 95% CI of adverse birth outcomes between the two comparison groups. In all comparisons, the differences were considered statistically significant at *p* < 0.05.

### Ethical consideration and consent to participate

Ethical approval was obtained from the Debre Markos University, School of Medicine's ethical review committee on 20 January 2019. Permission was also obtained from the concerned bodies of East Gojjam. To protect confidentiality, no personal identifier was recorded in the questionnaire, and the recorded data were not accessed by a third person. The study subjects were given all the information they needed to participate in the study through an informed consent form, which was read to those not able to read Amharic by the interviewer. The consent form was signed or marked with the thumbprint of the subject and signed by the interviewer and investigator. Only those who gave their consent participated in the study.

## Results

### Study participants' characteristics by the dietary diversity score

**Figure 1:**
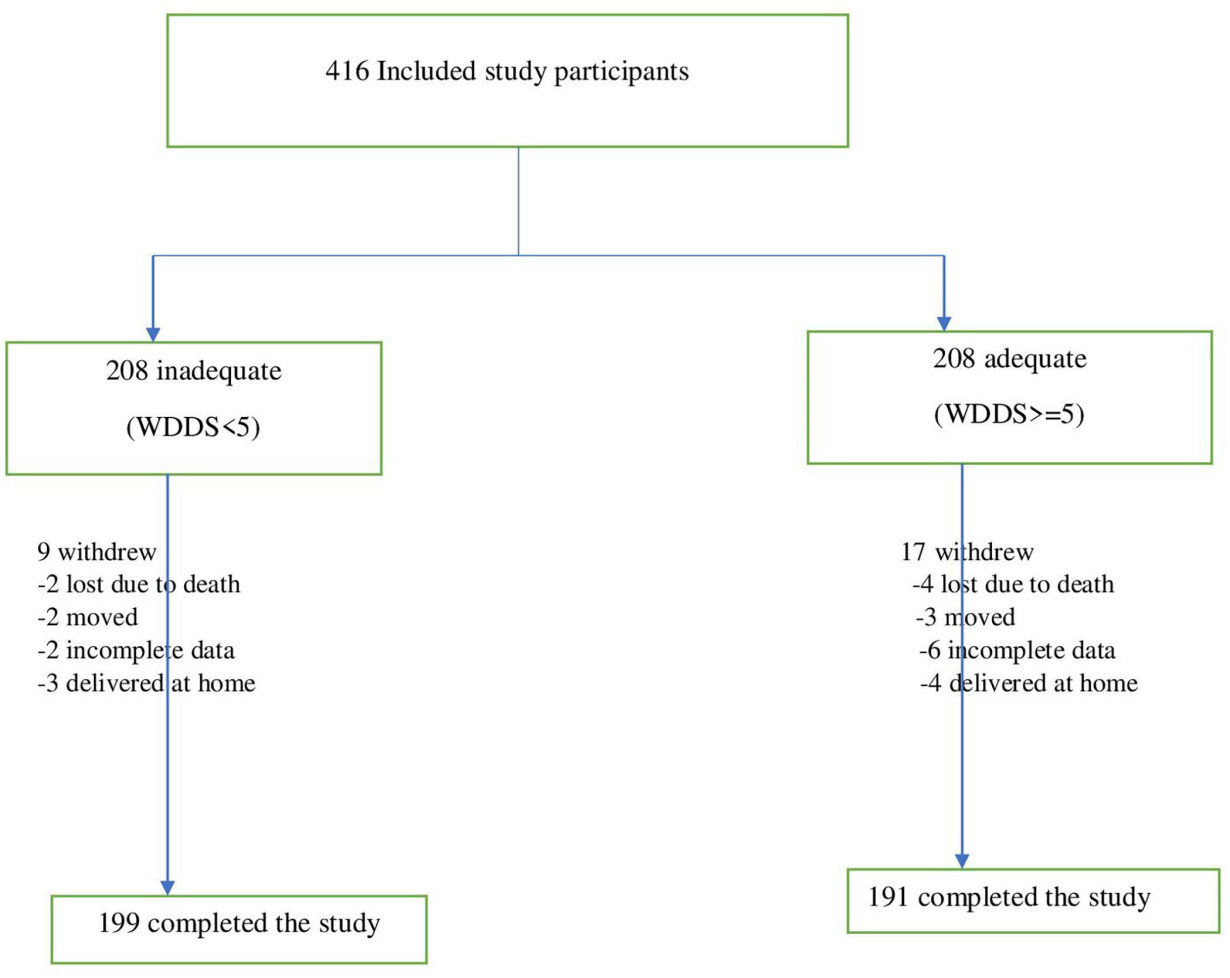
Revealed cohort of pregnant mothers follow-up procedure at east Gojjam zone, northwest Ethiopia, 2019/2020.

Out of 416, 390 study participants completed the follow-up, making a response rate of 94%. Twenty-six were withdrawn from the study and the major causes identified for loss to follow-up were due to death and movements (11 study participants), participants having incomplete data (eight participants), and delivery at home (seven study participants), and thus, the general dropout rate was 6%. Out of the total participants who finalized the study, 199 (51%) were inadequate diet diversity study participants (consumed less than or equal to four food groups) and 191 (49%) were adequate diet diversity study groups (consumed greater or equal to five food groups) (Figure 1).

The majority of study participants were literate, housewives in occupation, married, and orthodox Christians living in urban areas. Most of the study participants' gestational age at enrolment was 12 to 14 weeks (73.1%) (Table 1). The mean age of the study participants was 27 years with an SD of 4.53. The mean mid-upper arm circumference of the study participants was 24.5 cm with an SD of 1.76. Most study participants were gravida 3 and above and para 2. Seven women from non-diet diversity and eight women from diet diversity had a history of stillbirth. Five participants from non-diet diversity and also five participants from diet diversity had a history of abortion. One participant with inadequate diet diversity and 12 (6.3%) participants with adequate diet diversity had a history of alcohol consumption. There were no participants who had a history of smoking.

**Table 1:** Baseline demographic, socio-economic, and anthropometric characteristics of the study participants in the east Gojjam zone, northwest Ethiopia, 2019/2020.

**Variables**	**Coding categories**	**WDDS**		***p*-value**
		**Inadequate No (%)**	**Adequate No (%)**	
Age category of participants	≤ 27 years	125 (54.6%)	104 (45.4%)	0.094
	>27 years	74 (46%)	87 (54%)	
Educational status	No education	17 (17.2%)	82 (82.8%)	< 0.0001
	Non-formal education	8 (42.1%)	11 (57.9%)	
	Primary education	62 (66%)	32 (34%)	
	Secondary education	57 (62.6%)	34 (37.4%)	
	Diploma and above	55 (63.2%)	32 (36.8%)	
Occupation status	House wife	88 (57.5%)	65 (42.5%)	< 0.0001
	Farmer	15 (16.7%)	75 (83.3%)	
	Employed	46 (66.7%)	23 (33.3%)	
	Merchant	32 (72.7%)	12 (27.3%)	
	Daily laborer	15 (53.6%)	13 (46.4%)	
	Others	3 (50%)	3 (50%)	
Marital status	Single	4 (44.4%)	5 (55.6%)	0.35
	Currently married	193 (50.9%)	186 (49.1%)	
	Widowed	2	0	
HH size	≤ 4	177 (59.6%)	120 (40.4%)	< 0.0001
	>4	21 (23.3%)	69 (76.7%)	
Religion	Orthodox	197 (51.7%)	184 (48.3%)	0.16
	Others	1	4	
Monthly income	≤ 3445.34 Ethiopian birr	116 (72%)	45 (28%)	0.006
	≥3445.35 Ethiopian birr	61 (56%)	48 (44%)	
Residence	Rural	45 (30.6%)	102 (69.4%)	< 0.0001
	Urban	154 (63.4%)	89 (36.6%)	
Gestational age at enrollment/1^st^ ANC Visit	< 12wks	6	0	< 0.0001
	12-14wks	95 (33.3%)	190 (66.7%)	
	>14wks	98 (99%)	1	
MUAC	≤ 25cm	115 (44.2%)	145 (55.8%)	< 0.0005
	>25cm	84 (64.6%)	46 (35.4%)	
Gravida	Gravida 1	69 (54.3%)	58 (45.7%)	0.001
	Gravida 2	73 (61.9%)	45 (38.1%)	
	Gravida 3 and above	57 (39.3%)	88 (60.7%)	
Para	Para 0	74 (55.2%)	60 (44.8%)	< 0.0001
	Para 1	72 (62.6%)	43 (37.4%)	
	Para 2	53 (37.6%)	88 (62.4%)	

WDDS, Women's Dietary Diversity Score; HH, Household; ANC, Antenatal care; MUAC, Mid-upper arm circumference.

### Food items consumed

**Figure 2:**
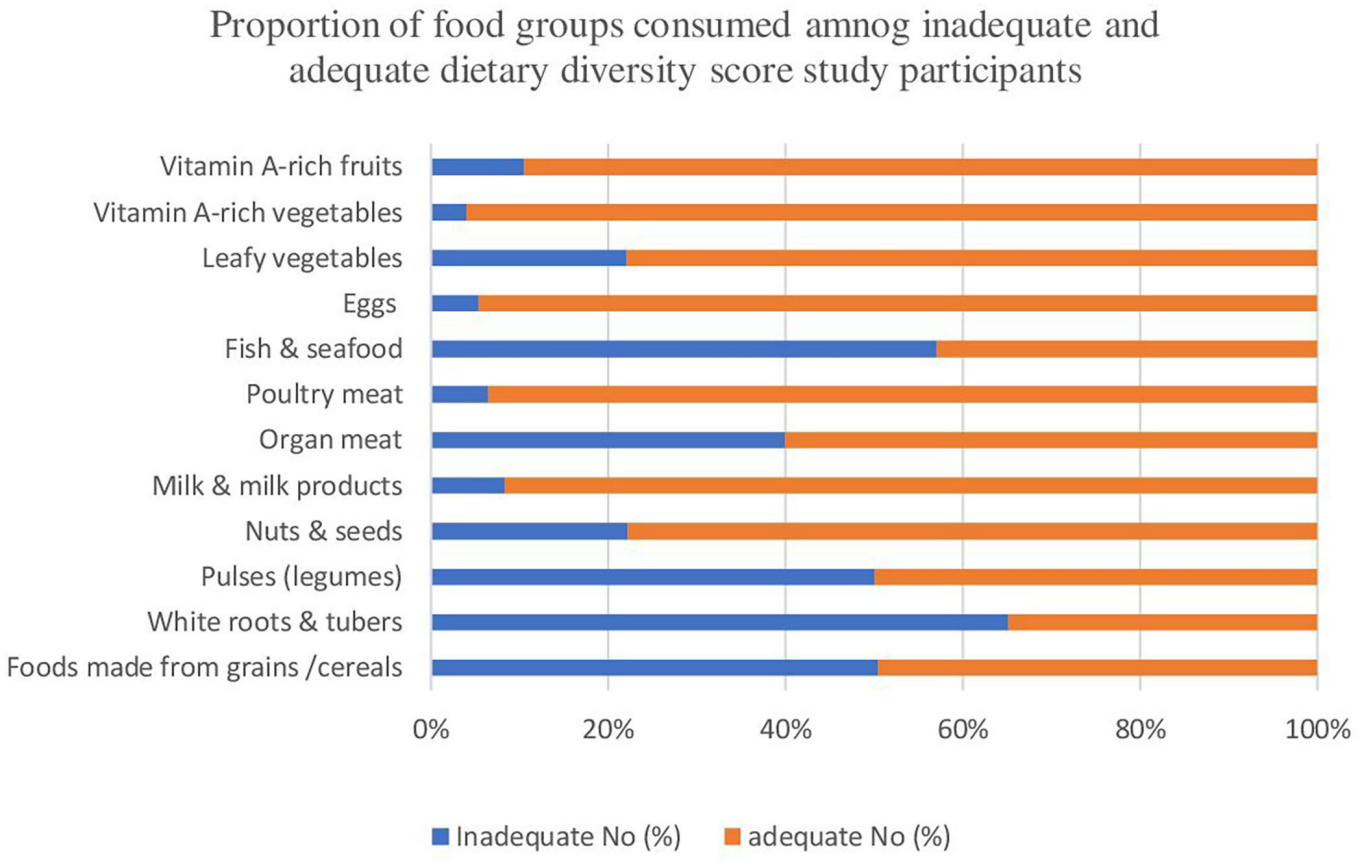
Revealed proportion of food groups consumed by diet diversity and non-diet diversity study participants in the east Gojjam zone, northwest Ethiopia, 2019/2020.

Out of the 10 food groups, almost all of the participants uniformly consumed foods made from grains/cereals (99% of participants) and pulses/legumes (98% of participants). But the adequate intake groups of participants were taking more dairy products, poultry meat, eggs, leafy vegetables, vitamin-A-rich vegetables, and fruits. The least consumed food groups by both study groups were nuts and seeds, organ meat, and fish and seafood (Table 2 and Figure 2). The mean dietary diversity score was 4.08 with an SD of 1.64 out of 10 maximum score. A significant difference in consumption of each food type was observed among diet diversity and non-diet diversity study participants based on the chi-square test.

**Table 2:** Food groups consumed by the study participants within 24 h of the interview in the east Gojjam zone, northwest Ethiopia, 2019/2020.

**Variables**	**Coding categories**	**WDDS**		***p*-value**
		**Inadequate Frequency**	**Adequate Frequency**	
Foods made from grains /cereals	Yes	195 (98%)	191 (100%)	0.049
	No	4 (2%)	0	
White roots and tubers	Yes	43 (21.6%)	23 (12%)	0.012
	No	156 (78.4%)	168 (88%)	
Pulses (legumes**)**	Yes	192 (96.5%)	191 (100%)	0.009
	No	7 (3.5%)	0	
Nuts and seeds	Yes	4 (2%)	14 (7.4%)	0.012
	No	195 (98%)	176 (92.6%)	
Milk and milk products	Yes	7 (3.5%)	77 (40.3%)	< 0.0001
	No	191 (96.5%)	114 (59.7%)	
Organ meat	Yes	2 (1%)	3 (1.6%)	0.62
	No	197 (99%)	188 98.4%)	
Poultry meat	Yes	7 (3.5%)	101 (52.9%)	< 0.0001
	No	192 (96.5%)	90 (47.1%)	
Fish and seafood	Yes	4 (2%)	3 (1.6%)	0.74
	No	195 (98%)	188 (98.4%)	
Eggs	Yes	4 (2%)	69 (36.1%)	< 0.0001
	No	195 (98%)	122 (63.9%)	
Leafy vegetables	Yes	49 (24.6%)	172 (90.1%)	< 0.0001
	No	150 (75.4%)	19 (9.9%)	
Vitamin A-rich vegetables	Yes	4 (2%)	95 (49.7%)	< 0.0001
	No	195 (98%)	96 (50.3%)	
Vitamin A-rich fruits	Yes	15 (7.5%)	127 (66.5%)	< 0.0001
	No	184 (92.5%)	64 (33.5%)	

**WDDS**, Women's Dietary Diversity Score.

### Energy, macronutrients, and micronutrients consumed analysis results

We have analyzed the mean values of energy, water, macronutrients, and micronutrients consumed within 24 h among the two comparison study groups (diet diversity vs. non-diet diversity). Almost all of the mean calculated values were significantly different between the two study groups (Table 3). Our finding showed that the mean energy value of the participants was significantly higher among dietary diversified (WDDS ≥ 5) groups compared to those non-dietary diversified study groups (WDDS < 5) and the variability is explained by 77.8%. A significantly higher mean water was consumed in the dietary diversity group compared to the non-diet diversity group with an explained variance of 80.7%. From the analyzed macronutrients, significantly higher consumption of protein was observed in the dietary diversity groups compared to no dietary diversity groups with 81.3% explained variability. A significantly higher consumption of fat was noticed among the dietary diversified group compared to the non-dietary diversified group with an explained variance of 80.3%. A significantly higher carbohydrate, fiber, PUFA, and cholesterol intake was observed among the dietary diversified groups compared to the non-dietary diversified group with an explained variance of 79.9, 71.1, 70.5, and 81.9%, respectively. More importantly, from analyzed micronutrients, significantly higher vitB1 (50%), vitB2 (36.2%), vitB6 (50.6%), folic acid (82.7%), sodium (82.5%), potassium (79.1%), calcium (78%), magnesium (83.6%), phosphorus (84.1%), iron (72.2%), and zinc (73.3%) consumption were obtained among dietary diversity group compared to non-dietary diversity group. On the other hand, our analysis found significantly higher consumption of vitamin A, carotene, and vitamin C in the non-dietary diversity group with an explained variance of 80.5, 69.9, and 80.7, respectively. Statistically significant differences observed between the two comparison groups were not lost in any of the variables analyzed when adjusted for socioeconomic characteristics and obstetric history of participants, telling us that the differences were not influenced by confounders.

**Table 3:** Consumed energy, macronutrients, and micronutrients by diet diversity of study participants in the east Gojjam zone, northwest Ethiopia. 2019/2020.

**Energy and nutrients**	**Inadequate (WDDS < 5)**	**Adequate (WDDS≥5)**	***p*-value**	**Eta squared value**
	**Mean (SD)**	**Mean (SD)**		
Energy (kcal)	1443.7 (104.02)	1877.8 (218.08)	< 0.0001	0.78
Water (g)	555.8 (32.51)	595.2 (27.78)	< 0.0001	0.81
**Macronutrients**				
Protein (g)	108.4 (6.07)	138.7 (7.61)	< 0.0001	0.81
Fat (g)	52.8 (9.01)	67.5 (7.05)	< 0.0001	0.80
Carbohydrate (g)	135.8 (13.77)	180.4 (11.59)	< 0.0001	0.79
Dietary fiber (g)	25.1 (11.46)	29.5 (8.90)	< 0.0001	0.71
PUFA (g)	11.6 (6.59)	14.3 (8.79)	0.001	0.71
Cholesterol (mg)	360.9 (134.47)	406.9 (128.33)	0.001	0.82
**Micronutrients**				
Vit. A (μg)	1817.6 (230.13)	1334.3 (102.67)	< 0.0001	0.81
Carotene (mg)	8.0 (3.09)	5.9 (2.06)	< 0.0001	0.69
Vit. E (mg)	11.5 (4.95)	11.6 (6.85)	0.826	0.75
Vit. B1 (mg)	1.5 (0.22)	2.0 (0.36)	< 0.0001	0.5
Vit. B2 (mg)	1.4 (0.14)	1.7 (0.64)	< 0.0001	0.36
Vit. B6 (mg)	2.4 (0.41)	3.1 (0.51)	< 0.0001	0.51
Folic acid (μg)	226.5 (15.61)	267.3 (14.77)	< 0.0001	0.83
Vit. C (mg)	56.3 (10.06)	47.9 (9.36)	< 0.0001	0.81
Sodium (mg)	315.6 (21.99)	368.3 (27.19)	< 0.0001	0.83
Potassium (mg)	2564.9 (268.89)	2937.6 (165.58)	< 0.0001	0.79
Calcium (mg)	367.9 (33.77)	461.4 (59.34)	< 0.0001	0.78
Magnesium (mg)	343.9 (33.59)	481.3 (46.87)	< 0.0001	0.84
Phosphorus (mg)	1400.4 (108.36)	1886.3 (67.28)	< 0.0001	0.84
Iron (mg)	16.3 (2.68)	24.7 (4.95)	< 0.0001	0.72
Zinc (mg)	16.5 (5.18)	24.1 (7.54)	< 0.0001	0.73

WDDS, Women's Dietary Diversity Score; PUFA, Poly Unsaturated Faty Acids; SD, Standard deviation.

### Pregnancy outcomes

The overall magnitude of low birth weight, preterm birth, and stillbirth in the study area, respectively, was 41, 37.9, and 4.1% (Table 4). In addition, the mean birth weight of the newborns born from mothers of inadequate intake was 2.34 kg, with an SD of 0.24, and those born from mothers of adequate intake were 2.8 kg, with an SD of 0.17 (*p* < 0.001). The mean gestational age at birth of those newborns delivered from mothers having inadequate intake was 35.5 weeks, with an SD of 2.9, and those delivered from mothers having adequate intake was 38 weeks, with an SD of 0.86 (*p* < 0.001) (Table 5).

**Table 4:** Pregnancy outcomes of the study participants in the east Gojjam zone, northwest Ethiopia, 2019/2020.

**Variables**	**Coding categories**	**WDDS**		***p*-value**
		**Inadequate No (%)**	**Adequate No (%)**	
Birth weight in kg	< 2.5kg (low birth weight)	147 (91.9%)	13 (8.1%)	< 0.0001
	≥2.5kg (normal birth weight)	52 (22.6%)	178 (77.4%)	
Gestational age at birth in weeks	< 37weeks (preterm)	136 (91.9%)	12 (8.1%)	< 0.0005
	≥37weeks (full term)	63 (26%)	179 (74%)	
Birth	Live birth	185 (49.5%)	189 (50.5%)	0.003
	Still birth	14 (87.5%)	2 (12.5%)	

WDDS, Women's Dietary Diversity Score.

**Table 5:** The mean birth weight and preterm birth of the study participants in the east Gojjam zone by their dietary diversity score status, east Gojjam zone, northwest Ethiopia, 2019/2020.

	**Inadequate group (*n =* 199)**		**Adequate group (*n =* 191)**			
**Selected outcome variables**	**Mean (SD)**	**95% CI**	**Mean (SD)**	**95% CI**	**Independent sample *t*-test (p)**	**Effect size (Cohen's d)**
Birth weight in kg	2.34 (0.24)	2.31–2.38	2.75 (0.17)	2.73–2.78	< 0.001	2.01
Gestational age at birth in weeks	35.52 (2.91)	35.09–35.87	38.02 (0.86)	37.89–38.13	< 0.001	1.32

SD, Standard deviation.

### Logistic regression analysis results

Our log-binomial regression analysis results revealed a statistically significant association between dietary diversity score during pregnancy and birth outcomes such as low birth weight and preterm birth. The risk of low birth weight of those newborns delivered from mothers who had an inadequate dietary diversity score while pregnant was about six times higher (ARR = 95% CI; 6.4, 3.4,12) as compared to those born from mothers who had adequate dietary diversity while pregnant. Similarly, the risk of preterm birth among those newborns delivered from those mothers with an inadequate dietary diversity score during pregnancy was about six times higher (ARR = 95% CI; 6.3, 3.3, 11.95) as compared to those born with adequate dietary diversity score mothers. Our study did not find a statistically significant association between dietary diversity score during pregnancy and the risk of stillbirth (ARR = 95% CI; 1.08, 0.2, 5.79) in newborns (Table 6).

**Table 6:** Log-binomial regression analysis result of pregnancy and birth outcomes of study participants by their dietary diversity score status, east Gojjam zone, northwest Ethiopia.

	**Selected birth outcomes**			
**Variables and coding categories**	**Birth weight category**		(ARR)^*^with 95% CI	***P*-value**
	** < 2.5 kg**	**≥2.5 kg**		
**Dietary diversity score status**				
Inadequate dietary diversity intake	147	52	6.4 (3.4, 12)	< 0.001
Adequate dietary diversity intake	13	178	1	
	**Gestational age at delivery**		**(ARR)** ^*^ **with 95% CI**	***P*** **-value**
	<**37weeks**	≥**37 weeks**		
**Dietary diversity score status**				
Inadequate dietary diversity intake	136	63	6.3 (3.3, 11.95)	< 0.001
Adequate dietary diversity intake	12	179	1	
	**Birth status**		**(ARR)** ^*^ **with 95% CI**	***P*** **-value**
	**Still birth**	**Live birth**		
**Dietary diversity score status**				
Inadequate dietary diversity intake	14	185	1.08 (0.20, 5.79)	0.926
Adequate dietary diversity intake	2	189	1	

* Adjusted for age, educational status, occupational status, abortion history, stillbirth history, MUAC, substance use history, and monthly HH income differences of the study groups. ARR, Adjusted Relative Risk.

## Discussion

Our analysis result showed that the most uniformly consumed food groups in the last 24 h by pregnant women in the first and second trimesters thereafter were grains and legumes. This may be because, in Ethiopia, the most widely produced agricultural products were grains and legumes (26). As is known, the most favorite and usually consumed processed food in Ethiopia is injera, which is made of a tiny rounded grain called teff. More importantly, most of the grains in Ethiopia are relatively cheap to purchase and consume as the majority of our study participants were urban residents (62%). In agreement with the current finding, previous studies reported that foods rich in protein, nutrient-rich vegetables, and fruits are less accessible to low-income people (27, 28). Our study found a relatively higher proportion of consumption of vegetables and fruits by pregnant women in diet diversity groups. This may be because vegetable and fruit types consumed may be easily cultivated by farmers compared to cultivating others as the majority of diet-diversified study participants were farmers by occupation or may be cheap to purchase and consume by non-farming study participants. Our study is in agreement with the previous reports, which found more consumption of fruits and vegetables by diet-diversified women (29, 30). A higher proportion of dairy products, poultry meat, and eggs was consumed by the diet diversity study participants. This may be because most diverse diet groups in this study resided in the rural area, which is more appropriate in the setting for farming, the rearing of cows for milk and its products, huge production of poultry for meat purposes, and the production of poultry and other eggs compared to the urban setting in Ethiopia. Consumption of nuts and seeds by our study participants was lower, which is in agreement with a previous study (31). In the same way, consumption of fish and seafood was low among our study participants. This may be due to the little practice of production which leads to low access to the market, which in turn leads to low consumption of sea foods in Ethiopia as well as in the continent of Africa (32–35), even though there are many huge rivers and lakes in Ethiopia and in the continent of Africa as well.

Our analysis showed that diet diversity was significantly associated with energy, water, and consumption of macronutrients and micronutrients. Significantly higher energy and water consumption values were observed among diet-diversified groups. In most of the cases, significantly higher macronutrient and micronutrient intake values were observed among the diet diversity study participants. Similar to our study, in the previous study, it was noticed that diet diversity had a direct association with nutrient intake and adequacy (36, 37). More importantly, previous studies also reported significant associations between diet diversity and different nutrients and energy consumption levels (38, 39). Significantly higher energy value was observed among diet-diversified study participants, in agreement with previous studies (18, 40). In agreement with our study, a previous study found significantly higher energy, protein, and zinc in dietary-diversified women (41). In contrast to our study, this study found significantly higher vitamin C consumption among diet-diversified women (41). This all may be because the consumption of more foods or more food groups may serve as a source of a variety of macronutrients and micronutrients. In addition, the significant difference in macronutrients and micronutrients is may be due to the consumption of a higher proportion of animal-sourced foods by the study participants of the diet diversity group, as animal-sourced foods are rich in macronutrients and micronutrients (42).

The current prospective cohort study was conducted in an urban and rural setting in northwest Ethiopia. The study enrolled pregnant women from their first ANC visit (first and second trimester) and followed until delivery to investigate the association between the dietary diversity of pregnant women and the risk of LBW, PTB, and stillbirth during pregnancy.

After adjustment for baseline characteristics, the present study revealed that pregnant women in the inadequate WDDS group were at higher risk of LBW and PTD as compared with their counterparts in the adequate WDDS group. This could be because the high DDS diet during pregnancy might contain several essential nutrients that are important in the growth and development of the fetus and newborn (43). Previous epidemiological and randomized control trial studies also found that better dietary practice during pregnancy was associated with improved birth outcomes including birth weight and preterm birth (13, 14, 44). On the other hand, previous findings showed that inadequate intake of foods and nutrients might lead to a wider range of adverse fetal outcomes, extending from premature birth and congenital anomalies such as neural tube and other neurodevelopment defects to death (45, 46).

After adjustment of possible covariates, the present study found that the risk of LBW in the inadequate group was six times higher compared to those born from the adequate dietary diversity score group. Our finding is consistent with that of previous studies (12, 47, 48). In contrast, a recent randomized controlled trial study in India reported that increased consumption of dairy, fruits, and green leafy vegetables before and during pregnancy through a specially formulated snack did not affect birth weight (15). Similarly, another previous study reported that there was no association between maternal macronutrient intake during pregnancy and infant birth weight (14). The variances in these studies can be attributed to differences in study designs, sample size, socioeconomic status, and analysis approaches.

The present study found that the relative risk of preterm birth among mothers with inadequate dietary diversity scores was six times higher than those newborns from mothers with adequate dietary diversity scores. This finding is consistent with that of previous findings conducted elsewhere (9, 12). The prevalence of preterm birth in African countries is the highest because of the largely deprived nutritional status (49).

The major limitation of our study may be considering only prenatal food consumption, as pre-pregnancy nutritional and health status, especially in the periconceptional period, are vital for both the mother's health and fetal development. This study established an association between diet diversity during gestation and low birth weight as well as diet diversity during gestation and preterm birth but did not establish causality among the aforementioned variables. Thus, the interpretation of our study findings should be cautious of these limitations. But this study has strengths. The first is being prospective in nature, as it may highly decrease the chance of missing necessary data. Also, to the best of our knowledge, our study is the first to reveal, especially the energy, water, and different macronutrient and micronutrient consumption by diet diversity category among pregnant women in poor socioeconomic settings such as in Ethiopia.

## Conclusion

Maternal dietary intake plays a crucial role in influencing the growth of offspring and birth outcomes. Diet diversity intake is a modifiable risk factor of public health importance in the effort to prevent adverse birth outcomes, particularly among developing/low-income populations.

Overall, the present study found a large magnitude of adverse birth outcomes, especially low birth weight and preterm birth. Inadequate intake of dietary diversity during pregnancy significantly increased the rate of low birth weight and preterm birth. Thus, we recommend the concerned body to work on improving the feeding practices of pregnant mothers in the study area. More importantly, the association between the WDD scores during pregnancy and fetal death needs further investigation.

## Data availability statement

The original contributions presented in the study are included in the article/supplementary material, further inquiries can be directed to the corresponding authors.

## Ethics statement

The studies involving human participants were reviewed and approved by the Ethical approval was obtained from Debre Markos University, School of Medicine ethical review committee on 20/1/2019. The patients/participants provided their written informed consent to participate in this study.

## Author contributions

AW: conception of the research protocol, study design, literature review, data collection, data entry, data analysis, interpretation, and drafting of the manuscript. BT: data collection, data entry, data analysis, and manuscript writing. AM: literature review, data collection, data entry, data analysis, and manuscript writing. AN: data collection, data entry, data analysis, interpretation, and manuscript writing. All authors have read and approved the manuscript.

## Funding

The authors received financial support for this research from Debre Markos University.

## Conflict of interest

The authors declare that the research was conducted in the absence of any commercial or financial relationships that could be construed as a potential conflict of interest.

## Publisher's note

All claims expressed in this article are solely those of the authors and do not necessarily represent those of their affiliated organizations, or those of the publisher, the editors and the reviewers. Any product that may be evaluated in this article, or claim that may be made by its manufacturer, is not guaranteed or endorsed by the publisher.

## Abbreviations

ARR: Adjusted Relative Risk
CI: Confidence Interval
FAO's: Food and Agricultural Organization's (FAO's)
LBW: Low Birth Weight
PTD: Preterm Delivery
SD: Standard deviation
WDDS: Women's Dietary Diversity Score.
